# Prevalence and Characterization of *Staphylococcus aureus* Isolated From Pasteurized Milk in China

**DOI:** 10.3389/fmicb.2019.00641

**Published:** 2019-04-02

**Authors:** Jingsha Dai, Shi Wu, Jiahui Huang, Qingping Wu, Feng Zhang, Jumei Zhang, Juan Wang, Yu Ding, Shuhong Zhang, Xiaojuan Yang, Tao Lei, Liang Xue, Haoming Wu

**Affiliations:** ^1^Guangdong Institute of Microbiology, State Key Laboratory of Applied Microbiology Southern China, Guangdong Provincial Key Laboratory of Microbial Culture Collection and Application, Guangdong Open Laboratory of Applied Microbiology, Guangzhou, China; ^2^School of Bioscience and Bioengineering, South China University of Technology, Guangzhou, China; ^3^College of Food Science, South China Agricultural University, Guangzhou, China; ^4^Department of Food Science and Technology, Jinan University, Guangzhou, China

**Keywords:** *Staphylococcus aureus*, pasteurized milk, risk assessment, virulence genes, antibiotic resistance, biofilm formation, MLST, *spa* typing

## Abstract

*Staphylococcus aureus* is one of the most important food-borne pathogens globally. It produces various toxins and invasive enzymes and can be found in numerous food products. Milk is an important source of staphylococcal food poisoning. After pasteurization, this microorganism or its enterotoxins might still remain in pasteurized milk. Therefore, this study was to investigate the contamination of *S. aureus* in 258 pasteurized milk from 39 cities of China. The prevalence and levels of *S. aureus* in these samples as well as antibiotic susceptibility profiles, virulence genes, biofilm formation, and biofilm related genes, *spa* typing and MLST were used to determine the characterization among the isolates. It was found 3.9% of samples were detected *S. aureus* in 8 of 39 cities in China. The contaminated level were not very excessive which showed the MPN values of the most positive samples (9/10) were less than 1 MPN/g. All pasteurized milk-related *S. aureus* isolates have ability to produce biofilm and harbored *icaA, icaD, eno*, *clfA, clfB, fnbA, fnbB, fib* genes, other biofilm related genes *icaC* were showed in 91.7% of isolates and *cna* gene were showed in 50%, except *bap* gene which were free in all isolates. The antibiotic susceptibility test showed that all isolates were resistant or intermediate-resistant to different concentrations of the antibiotics. Furthermore, 75.0% of the isolates were resistant to three or more antibiotic classes, which indicated multidrug resistance. The isolates had virulence potential, which showed 66.7% (8/12) of the isolates carried one or more virulence-associated genes. Molecular typing by MLST and *spa* typing enabled classification of these isolates into a total of 11 sequence types (STs) and *spa* types, which indicated high genetic diversity. Most of these types were related to various clinical *S. aureus* infections. Thus, the findings of this study reflect the potential risk of *S. aureus* infection in China. Our study also provides comprehensive analysis of the prevalence of *S. aureus* in pasteurized milk and helps ensure more accurate treatment of human infection with effective antibiotics.

## Introduction

*Staphylococcus aureu*s is a pathogen associated with serious community and hospital-acquired diseases. It has low nutritional requirements and widely exists in nature. In China, microbial food poisoning accounted for 53.7% of food poisoning emergencies in 2015. Of which, *S. aureus* was an important pathogenic factor in these cases (Wu et al., 2018a). In the United States, *S. aureus* causes approximately 241,000 cases of food poisoning each year (Scallan et al., 2011; Kadariya et al., 2014). This foodborne pathogen is considered as one of the world’s leading causes of disease outbreaks related to food consumption, being responsible for a variety of manifestations and diseases (Jamali et al., 2015).

*Staphylococcus aureus* produces a variety of toxins and invasive enzymes such as staphylococcal enterotoxins (SEs), hemolysins, Panton-Valentine leukocidin (PVL), toxic shock syndrome toxin-1 (TSST-1), plasma coagulase, and deoxyribonuclease (Spanu et al., 2012). Differentiation between virulent and non-virulent strains is significant for evaluating the potential implications of the presence of this microorganism for food safety and public health. In which, SEs are active at concentrations ranging from high nanogram to low microgram quantities (Larkin et al., 2009) and are resistant to conditions (heat treatment, low pH) that retaining their activity in the digestive tract after ingestion (Evenson et al., 1988; Argudín et al., 2010). In addition, TSST-1 is a superantigenic exotoxin that causes toxic shock syndrome and PVL is a bacteriophage-encoded bicomponent leukotoxin that is in some strains of *S. aureus* and plays a key role in leukocytolysis and tissue necrosis (Shallcross et al., 2013). *S. aureus* often develops antibiotic resistance. Isolation of single- or multiple-drug resistant *S. aureus* (MRSA) strains from food, the environment, and clinics has been constantly reported (Gould et al., 2012; Rasigade et al., 2014). The ability of *S. aureus* to form biofilms helps the bacterium to survive in hostile environments within the host and is considered to be responsible for chronic or persistent infections (Costerton et al., 1999). The ability of some strains to synthesize biofilms could increase their pathogenicity since established biofilms can tolerate antimicrobial agents, thus making the bacterium extremely difficult to eradicate (Zmantar et al., 2010).

Nowadays, molecular typing methods are crucial in epidemiological investigations of food processing and enhance the resolution of surveillance (Wu et al., 2015). Of various subtyping approaches, multilocus sequence typing (MLST) is a widely accepted method of DNA sequence based typing that based on analysis of relatively conserved genes that encode essential proteins. For *S. aureus*, the level of discrimination provided by MLST is sufficient to provide a relatively detailed picture of the global dissemination of the organism (Saunders and Holmes, 2007). *spa* typing is another efficient typing method for *S. aureus*, it based on sequencing of the polymorphic X region of the protein A gene (*spa*) (Hallin et al., 2009). Similar to MLST, it suggests that *spa* typing is suitable for epidemiology and evolutionary investigations based on studies of European and international isolates (Wu et al., 2018b).

Milk is an important source of staphylococcal food poisoning. There are several foodborne outbreaks of *S. aureus* intoxications have been documented to be associated with consumption of contaminated milk (De Buyser et al., 1985; Miwa et al., 2001; Fetsch et al., 2014). In addition, raw milk and raw milk products are frequently contaminated with different types of *S. aureus* around the world (Tham et al., 1990; Rosengren et al., 2010; Yu et al., 2010; Jamali et al., 2015; Riva et al., 2015). Milk is a good substrate for *S. aureus* growth and enterotoxin production. Enterotoxins can retain their biological activity after pasteurization (Asao et al., 2003; Rall et al., 2008). In China, studies have reported that some *S. aureus* strains persist in powdered infant formula (Wang et al., 2012). Currently, only a few full-scale and systematic studies have been performed on *S. aureus* prevalence and contamination levels in pasteurized milk in China. This study aimed to investigate *S. aureus* contamination in pasteurized milk obtained from different Chinese cities. For this purpose, we analyzed the *S. aureus* prevalence and contamination levels, antibiotic susceptibility profiles, virulence genes, biofilm formation, biofilm-related genes, *spa* typing results, and multilocus sequence typing (MLST) results to characterize the isolates.

## Materials and Methods

### Sampling

From July 2011 to June 2016, a total of 258 pasteurized milk samples were collected from supermarkets, fairs, and farmers’ markets (Supplementary Table 1). The samples were obtained from 39 cities located in a total of 29 provinces and 2 directly controlled municipalities in China, which covered most of the provincial capitals of China (Supplementary Figure 1). The samples were placed in a cold box at approximately 4°C, tightly sealed with sterile plastic wrap, transported to an accredited laboratory, and subjected to microbiological analysis within 24 h.

### Isolation and Detection

The samples were qualitatively and quantitatively analyzed to detect *S. aureus*. For qualitative analysis, samples were examined according to GB 4789.30-2010 (National Food Safety Standards of China) with slight modification. The most probable number (MPN) method was used for quantitative analysis. Approximately 25 g of the food sample was homogenized in 225 mL tryptic soy broth with 10% sodium chloride (Huankai, Guangzhou, China). Subsequently, 1-mL, 0.1-mL, and 0.01-mL aliquots were transferred to tubes containing 9, 10, and 10 mL in triplicate with trypticase soy broth (Huankai) supplemented with 10% NaCl. The tubes were then incubated at 37°C for 48 h.

A loopful of enrichment broth culture (10 μL) was streaked onto chromogenic *S. aureus* agar plates (Huankai) and incubated at 37°C for 24 h. Of the colonies obtained, 1–4 pink colonies were purified on nutrient agar medium. The purified colonies were analyzed via the coagulase activity test involving freeze-dried rabbit plasma (Huankai), and the API STAPH test strips (bioMérieux, Marcy-l’Étoile, France) were then used. The MPN value was determined on the basis of the number of positive tube(s) in each of the three sets using the MPN table.

### Antimicrobial Susceptibility Testing

The Kirby–Bauer disk diffusion method was used to test antibiotic susceptibility, and diameter interpretations were based on the protocol specified in the guidelines of Clinical and Laboratory Standards Institute [CLSI] (2015). *S. aureus* ATCC 25923 and *Escherichia coli* ATCC 25922 were used as quality control organisms. All isolates were assessed for antimicrobial susceptibility to 24 antibiotics (Oxoid, United Kingdom): amoxicillin/clavulanic acid, ampicillin, cefepime, cefoxitin, penicillin G, ceftazidime, amikacin, gentamicin, kanamycin, streptomycin, chloramphenicol, clindamycin, erythromycin, telithromycin, ciprofloxacin, norfloxacin, tetracycline, linezolid, trimethoprim/sulfamethoxazole (1:19), rifampicin, quinupristin/dalfopristin, teicoplanin, nitrofurantoin, and fusidic acid. The CLSI zone diameter breakpoints were used to interpret the antimicrobial susceptibilities of the analyzed strains.

### *In vivo* Biofilm Formation

Biofilm production was quantified using a microtiter plate assay (MPA) described previously (Vasudevan et al., 2003), with slight modifications. *S. aureus* strains were individually grown at 37°C overnight in brain heart infusion broth (BHI). The overnight culture was diluted 1:100 in fresh BHI, and 200-μL aliquots of each prepared suspension were transferred into three wells of 96-well tissue culture treated polystyrene microplates (CELLSTAR^®^ Cell Culture Microplates, Greiner Bio-one, Frickenhausen, Germany). After cultivation at 37°C for 48 h, the wells were washed three times with 200 mL sterile phosphate-buffered saline (PBS, pH 7.4) and dried at room temperature. The adherent bacterial cells were fixed with 200 μL methanol for 15 min, and the plates were emptied and left to dry overnight. The adherent cells were then stained with 1% crystal violet for 10 min and washed twice with water. The dye bound to the adherent cells was dissolved with 150 mL 95% ethanol and optical density (OD) was measured at 590 nm with a spectrophotometer (SpectroStar Nano, BMG Labtech). Uninoculated wells containing BHI served as blanks. Blank-corrected absorbance values of *S. aureus* strains were used for reporting biofilm production. Isolates were considered biofilm producers when their OD values were three times greater than the standard deviation of the mean Dc. Additionally, isolates showing biofilm-producing ability were classified as weak (Dc < OD ≤ 2 × Dc), moderate (2 × Dc < OD ≤ 4 × Dc), or strong (OD > 4 × Dc) biofilm producers.

### DNA Extraction and PCR

After the isolates were cultivated for at 37°C 24 h in BHI, genomic DNA was extracted using a Genomic DNA Extraction kit (Magen Biotech, Guangzhou, China) according to the manufacturer’s instructions. The genomic DNA concentration was determined at 260 nm by using a NanoDrop-ND-1000 UV-Vis Spectrophotometer (Thermo Fisher Scientific, MA, United States). The *mecA/mecC* gene, which confers methicillin resistance to *S. aureus*, was detected by PCR performed using primers described previously (Perez-Roth et al., 2001; Stegger et al., 2012). PCR was also used to detect the presence of virulence genes. The detection of 18 SE genes (*sea, seb, sec, sed, see, seg, seh, sei, sej, sek, sel, sem, sen, seo, sep, seq, ser*, and *seu*), the *tsst* gene and the presence of *lukSF-PV* (*pvl*) genes was performed as previously described (Jarraud et al., 2002; Varshney et al., 2009). For adhesion genes (*clfA, clfB, fnbA, fnbB, fib*, and *cna*) and biofilm-producing genes (*icaA, icaD, icaC, bap*, and *eno*), PCR was performed according to previous studies (Zmantar et al., 2010; Artini et al., 2013; Salaberry et al., 2015; Pereyra et al., 2016). The primer sequences used in the PCR assays, expected amplified product sizes, and references are listed in Supplementary Table 2. The amplicons were stained with Goldview, loaded onto 1.5% agarose gels, electrophoresed at 120 V for 0.5 h, and visualized by a UV transilluminator gel imaging system (GE Healthcare, WI, United States). The images were saved as TIFF files for analysis.

### Molecular Typing

All the isolates were characterized by MLST and *spa* typing. The MLST analysis was based on DNA sequencing of the seven housekeeping genes *arcC, aroE, glpF, gmk, pta, tpi*, and *yqil*, as described previously (Enright et al., 2000). The primers spa-1113f (5′-TAA AGA CGA TCC TTC GGT GAG C-3′) and spa-1514r (5′-CAG CAG TAG TGC CGT TTG CTT-3′) were used for *spa* amplification (Shopsin et al., 1999). The PCR products were purified with a PCR purification kit (Qiagen, Genmany) and were sequenced in both directions with Big Dye fluorescent terminators on an ABI 3730XL sequencer (Applied BioSystems). For each MLST locus, an allele number was assigned to each distinct sequence variant, and a distinct sequence type (ST) number was attributed to each distinct combination of alleles for the seven genes. STs were determined using the *S. aureus* MLST database.^1^ Clonal complex (CC) analysis was performed using eBURST v.3 as previously described (Feil et al., 2004). The *spa* types were randomly assigned using the SpaServer website.^2^ Sequence Type Analysis and Recombinational Tests software (S.T.A.R.T. ver. 2; https://pubmlst.org/software/analysis/start2) was used to analyze the MLST data.

## Results

### Isolation and Identification of *S. aureus* From Pasteurized Milk

Of the 258 pasteurized milk samples, 10 samples were contaminated with coagulase-positive staphylococci, and 12 isolates obtained from these positive samples were confirmed as *S. aureus* by the API STAPH test. The qualitative and quantitative results for these positive samples are shown in Table 1. Overall, the mean prevalence of *S. aureus* was 3.9%, and the MPN values for the samples showing the highest levels (9/10) were less than 1 MPN/g. Positive samples were obtained from 8 of the 39 Chinese cities included (Supplementary Figure 1): 33.3% of the positive samples were from Zhanjiang and Xining cities, and 16.7% were obtained from the other six cities, respectively. Besides, among the different types of sampling source, the positive samples isolated from fairs was 4.2% (6/144) followed by farmer’s market (3.8%, 1/26) and supermarkets (3.4%, 3/88).

**Table 1:** Prevalence and contamination levels of *Staphylococcus aureus* in pasteurized milk samples from China.

Positive number	Positive sample	Sampling site	Source	Quantitative methods	Qualitative methods
				MPN values (MPN/g)	
1	ZJJ376	Zhanjiang	Fair	0.92	+
2	ZJC1758	Zhanjiang	Supermarket	<0.3	+
3	FZJ2233	Fuzhou	Fair	24	+
4	XMJ2333	Xiamen	Fair	0.36	+
5	LZN1142	Lanzhou	Farmer‘s market	<0.3	+
6	XNC3008	Xining	Supermarket	0.3	+
7	XNJ3032	Xining	Fair	0.3	+
8	XGJ3532	Hongkong	Fair	0.75	+
9	GYC3608	Guiyang	Supermarket	<0.3	+
10	HHHTJ4032	Huhehaote	Fair	0.74	+

### Antibiotic Resistance

Most *S. aureus* isolates showed resistance and intermediate resistance to different antibiotics concentrations. The resistance patterns of *S. aureus* to the tested antimicrobial agents are presented in Table 2. The phenotypic resistance profiles of the *S. aureus* isolates are as follows: ampicillin, 97.7%; penicillin G, 83.3%; erythromycin, 50.0%; kanamycin, 41.7%; telithromycin, 33.3%; clindamycin, 33.3%; gentamicin, 33.3%; tetracycline, 33.3%; norfloxacin, 25.0%; ciprofloxacin, 16.7%; amikacin, 16.7%; rifampicin, 16.7%; chloramphenicol, 8.3%; fusidic acid, 16.7%; ceftazidime, 8.3%; streptomycin, 8.3%; amoxicillin/clavulanic acid, 8.3%; trimethoprim/sulfamethoxazole (1:19), 8.3% and quinupristin/dalfopristin, 8.3%. All the isolates were susceptible to cefepime, cefoxitin, linezolid, and nitrofurantoin. The *mecA/mecC* gene was free in all *S. aureus* isolates. Combining with antibiotic resistance test, there was none of MRSA isolates identified. However, only one isolate was susceptible to all the antimicrobial agents tested, whereas two isolates were resistant to two antibiotics and 75.0% of isolates (9/12) were resistant to three or more classes of antibiotics which showed multi-drug resistance including one strain Sta1758 were resistant to more than 10 antibiotics.

**Table 2:** Antimicrobial susceptibility tests for *Staphylococcus aureus* isolates obtained from pasteurized milk samples from China.

	Antibiotics	*S. aureus* (*n* = 12)		
		No. of resistant strains (%)	No. of intermediate-resistance strains (%)	No. of susceptible strains (%)
β-Lactams	Amoxycillin/clavulanic acid	1 (8.3%)	–	11 (97.7%)
	Ampicillin	11 (97.7%)	–	1 (8.3%)
	Cefepime	0 (0%)	0 (0%)	12 (100%)
	Cefoxitin	0 (0%)	–	12 (100%)
	Penicillin G	10 (83.3)	–	2 (16.7%)
	Ceftazidime	1 (8.3%)	2 (16.7%)	9 (75.5%)
Aminoglycosides	Amikacin	2 (16.7%)	2 (16.7%)	8 (66.7%)
	Gentamicin	4 (33.3%)	–	8 (66.7%)
	Kanamycin	5 (41.7%)	2 (16.7%)	5 (41.7%)
	Streptomycin	1 (8.3%)	8 (66.7%)	3 (25.0%)
Phenicols	Chloramphenicol	1 (8.3%)	3 (25.0%)	8 (66.7%)
Lincosamides	Clindamycin	4 (33.3%)	1 (8.3%)	7 (58.3%)
Macrolides	Erythromycin	6 (50.0%)	1 (8.3%)	5 (41.7%)
	Telithromycin	4 (33.3%)	3 (25.0%)	5 (41.7%)
Fluoroquinolones	Ciprofloxacin	2 (16.7%)	5 (41.7%)	5 (41.7%)
	Norfloxacin	3 (25.0%)	3 (25.0%)	6 (50.0%)
Tetracyclines	Tetracycline	4 (33.3%)	–	8 (66.7%)
Oxazolidinones	Linezolid	0 (0%)	0 (0%)	12 (100%)
Ansamycins	Rifampicin	2 (16.7%)	1 (8.3%)	9 (75.0%)
Sulfonamides	Trimethoprim/sulfamethoxazole (1:19)	1 (8.3%)	0 (0%)	11 (97.7%)
Quinolones	Quinupristin/dalfopristin	1 (8.3%)	0 (0%)	11 (97.7%)
Glycopeptides	Teicoplanin	0 (0%)	2 (16.7%)	10 (83.3%)
Nitrofurantoins	Nitrofurantoin	0 (0%)	0 (0%)	12 (100%)
	Fusidic acid	2 (16.7%)	–	10 (83.3%)

### *In vitro* Biofilm Production and Presence of Biofilm-Related Genes

The MPA results showed that all the *S. aureus* strains could produce biofilms, including four isolates (33.3%, 4/12) showing moderate and eight isolates (76.7%, 8/12) showing strong biofilm formation (Table 3). Table 3 also provides the results for the presence of *S. aureus* adhesion and biofilm-associated genes. Overall, *icaA, icaD, eno*, *clfA, clfB, fnbA, fnbB*, and *fib* were found in all 12 isolates, whereas the *bap* gene was free in all isolates. In addition, *icaC* was present in 91.7% (11/12) of the isolates and the *cna* gene in 50% (6/12). Thus, most of the biofilm-producing genes and adhesion genes were present in these *S. aureus* strains isolated from the pasteurized milk samples.

**Table 3:** Biofilm-formation ability and biofilm-associated genes in *Staphylococcus aureus* isolates obtained from pasteurized milk samples.

No.	*S. aureus* isolates	Biofilm production assay	Biofilm production ability^a^	biofilm-producing genes					adhesion genes					
				*icaA*	*icaD*	*icaC*	*bap*	*eno*	*clfA*	*clfB*	*fnbA*	*fnbB*	*fib*	*cna*
1	Sta376	1.4715	+++	+	+	+	-	+	+	+	+	+	+	+
2	Sta1758	0.5147	++	+	+	+	-	+	+	+	+	+	+	-
3	Sta2233-0	1.2700	+++	+	+	+	-	+	+	+	+	+	+	-
4	Sta2333-0	0.8515	++	+	+	+	-	+	+	+	+	+	+	-
5	Sta1142	1.1548	+++	+	+	+	-	+	+	+	+	+	+	+
6	Sta3008C2	0.5417	++	+	+	+	-	+	+	+	+	+	+	+
7	Sta3032	0.6850	++	+	+	+	-	+	+	+	+	+	+	+
8	Sta3032C3	1.1167	+++	+	+	-	-	+	+	+	+	+	+	-
9	Sta3532	1.0995	+++	+	+	+	-	+	+	+	+	+	+	-
10	Sta3608	2.5659	+++	+	+	+	-	+	+	+	+	+	+	-
11	Sta4032B2	2.6347	+++	+	+	+	-	+	+	+	+	+	+	+
12	Sta4032B3	2.7753	+++	+	+	+	-	+	+	+	+	+	+	+

^a^*Quantification of biofilm formation by optical density (OD) analysis: (+++): strong biofilm producers (OD_*590*_ > 0.88), (++): moderate biofilm producers (0.44 > OD_*590*_ > 0.88), (+): weak biofilm producers (0.22 > OD_*590*_ > 0.44)*.

### Prevalence of Virulence-Associated Genes

**Figure 1:**
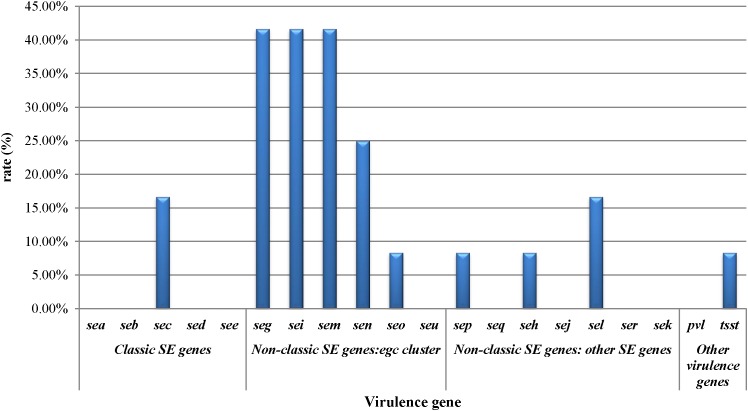
Staphylococcal toxin gene profiles of *Staphylococcus aureus* isolates obtained from pasteurized milk.

The prevalence of virulence-associated genes is shown in Figure 1. Of the *S. aureus* isolates analyzed, 66.7% (8/12) carried one or more virulence-associated genes. Of the 20 virulence genes investigated, *seg, sei*, and *sem* were most frequently detected which showed 41.7% of prevalence, respectively. *sea, seb, sed, see, seu, seq, sej, ser, sek*, and *pvl* were not detected in the *S. aureus* isolates. The prevalence of the other virulence genes is as follows: *sen* (25%), *sec* (16.7%), *sel* (16.7%), *seo* (8.3%), *sep* (8.3%), *seh* (8.3%), and *tsst* (8.3%). Staphylococcal enterotoxin genes can be divided into classic SE genes (*sea, seb, sec, sed*, and *see*), *egc* cluster genes (*seg, sei, sem, sen, seo*, and *seu*), and other novel SE genes (*sep, seq, she, sej, sel, ser*, and *sek*). From the classic SE genes, only two isolates showed the *sec* gene, that is 7.7% (2/26) prevalence in the detected genes. The *egc* cluster accounted for 73.1% (19/26) of the detected genes, and other novel SE genes accounted for 15.4%.

### Molecular Characterization of *S. aureus* Isolates

**Figure 2:**
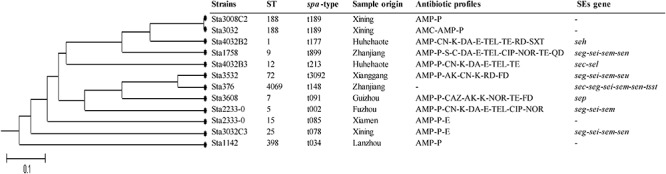
Unweighted pair group method with arithmetic mean (UPGMA) tree of the 7 multilocus sequence typing loci of *S. aureus* isolates obtained from pasteurized milk. This tree was generated using S.T.A.R.T. (version 2).

MLST analysis of the 12 isolates yielded 11 STs showing high genetic diversity. Except ST188 was found in two strains, the other STs were present in single strains which included ST1, ST5, ST7, ST9, ST12, ST15, ST25, ST72, ST398, and ST4069. Of these, ST4069 was a newly assigned ST, which found a single-locus variant (SLV) on the *aroE* gene (assigned as *aroE*-472). *spa* typing showed results similar to those of MLST. Eleven *spa* types were detected: t189 was found in two strains, and t177, t899, t213, t3092, t148, t091, t002, t085, t078, and t034 were found in single isolates. The phylogenetic tree based on the seven concatenated MLST sequences in Figure 2 shows the relatedness between these isolates. ST188 correlated well with t189, which was also found in city of Xining.

## Discussion

Pasteurization, or heat treatment, of milk is an important milestone in for public health and has contributed to a dramatic decline in many infectious diseases. However, outbreaks associated with pasteurized milk continue to occur (Ryan et al., 1987; Dalton et al., 1997; Ackers et al., 2000). In China, milk has become an important component of a balanced diet, and the annual per capita milk consumption increased from 9.23 kg in 1992 to 24.87 kg in 2007 (Qian et al., 2011; Liu and Wang, 2013). SFP outbreaks associated with milk have occurred occasionally (Dai et al., 2009). There were limited researches on the prevalence of *S. aureus* from pasteurized milk in China. In the current study, 3.9% of the samples showed the presence of *S. aureus*. Compare with other countries’ studies, the prevalence of *S. aureus* from pasteurized milk in our study is lower. In Brazil, Rall et al. (2008) collected 162 raw and pasteurized milk samples and found 20.4% of the pasteurized milk samples were positive for *S. aureus*. In 2006, Gündoğan et al. (2006) analyzed 180 raw milk, pasteurized milk, and ice cream samples; 56.6% of the pasteurized milk samples were positive for *S. aureus*. However, it is maybe attribute to the sample sizes, sample source or geographic locations, which showed most of these *S. aureus* were isolated from farm, whereas our samples were collected from supermarkets, fairs or farmer’s markets. Quantitative analysis by MPN showed that only one *S. aureus*–positive sample reached a level of 10 MPN/g. In China, a limit of 100 CFU/g (mL) has been proposed for *S. aureus* in milk and the highest fraction of samples with values that exceeded this limit was observed at the end of food shelf-life (Helwigh and Korsgaard, 2007). Thus, the currently available data, along with our study, indicate that levels of *S. aureus* in pasteurized milk in China are not very high.

Staphylococcal enterotoxins are responsible for most SFP outbreaks (Dinges et al., 2000; Loir et al., 2003). This could account for retention of biological activity after pasteurization. This study was investigated the 18 SE genes among pasteurized milk related *S. aureus* isolates and found that 66.7% of the isolates carried one or more SE genes, suggesting the risk of *S. aureus* contamination in pasteurized milk in China. This rate is higher than that reported in a previous study (Chao et al., 2015) in which the prevalence of 18 SE genes was studied in *S. aureus* isolates obtained from different origins in China, showing that 54.4% of those isolates harbored SE genes. Generally, the *sea* gene has been the most common gene in the SFP outbreak isolates tested, followed by the *sed* gene from classic SE genes (Kérouanton et al., 2007), but they were not detected in the current study. *sec* was the only classical SE gene detected in our study, which is the most commonly occurring SE in milk (Lindqvist et al., 2002; Scherrer et al., 2004; Aragon-Alegro et al., 2007). In 2002, an outbreak of community-acquired foodborne illness caused by MRSA was attributed to SEC-containing coleslaw, which was produced in the United States (Jones et al., 2002). In addition, we observed higher prevalence of *seg-sei-sem* among the isolates in our study, these SE genes belonged to the *egc* cluster, which is widely distributed in clinical isolates and acts as a putative nursery of enterotoxin genes (Jarraud et al., 2001; Wu et al., 2018b). Thus, the hazard posed by these isolates harboring SE genes should not be ignored.

In the current study, all *S. aureus* strains obtained from pasteurized milk samples could produce biofilms and showed moderate or strong biofilm production capability. In addition, various genes have been reported to be involve in biofilm formation, of these genes the *ica* group genes especially *icaA* and *icaD* (Arciola et al., 2001) and the other group such as *fnbA, fnbB, cna* and *eno* genes that encode MSCRAMMs proteins (Atshan et al., 2012) are noticeable. Most of these genes were present in the *S. aureus* strains isolated from our samples. This result were similar with the research by Atshan et al. (2012) who detected 9 MSCRAMM and 4 biofilm related genes in the MSSA and MRSA isolates from clinically patients by PCR method and found *fnbA, eno, ebpS, clfA, clfB, icaA, icaD, icaB*, and *icaC* genes were detected in 100% of isolates. Furthermore, *fib* and *cna* were detected in 90% and 46.6% MSSA isolates, respectively, which is in agreement with our result. In our study, the *bap* gene was free in all *S. aureus* isolates. Many studies have reported that the *bap* gene was not detected or very low rates in the solutes studied (Melchior et al., 2007; Szweda et al., 2012; Khoramrooz et al., 2016; Pereyra et al., 2016; Felipe et al., 2017). Although there was inconclusive evidence for the correlation between phenotypic tests and the detection of specific genes associated with adhesion and biofilm formation, the presence of *icaA, icaD, icaC, fnbA, clfA, clfB*, *fnbA, fnb*, and *fib* genes indicated a significant association with biofilm formation in this study. The biofilm production in isolates with negative results for any of the genes studied may be attributable to other biofilm-related genes.

Antimicrobial resistance was noted in pasteurized milk related *S. aureus* isolates. Most of the isolates were resistant to at least one antimicrobial agent. However, resistance to ampicillin and penicillin was higher than that reported in many previous studies involving *S. aureus* isolates from dairy samples (Rosengren et al., 2010; Jamali et al., 2015; Riva et al., 2015). We also observed that 25.0% of the isolates were resistant to multiple antibiotics such as ampicillin, gentamicin, kanamycin, clindamycin, erythromycin, and telithromycin. These were similar to the study by Jamali et al.’ investigation, which showed the resistance profiles of *S. aureus* isolates from raw milk and dairy products for tetracycline, penicillin, clindamycin, erythromycin, streptomycin, kanamycin, chloramphenicol, and gentamicin (Jamali et al., 2015). Furthermore, many isolates were found intermediate-resistant to the selected antibiotics in this study. The reduced antibiotic susceptibility of these antibiotics suggests that these bacteria may be exposed to such antimicrobials for a long time (Howden et al., 2010). Thus, it should draw public attention and controlled use of antimicrobials would limit the emergence of drug-resistant bacteria.

MLST and *spa* genotyping have shown a highly clonal population structure for *S. aureus* in many studies (Kanika et al., 2011; Fetsch et al., 2014; Basanisi et al., 2017). The *S. aureus* MLST database^3^ currently has 5024 different STs. However, some STs are specific to a particular source. For example, ST398 occurs in livestock-associated *S. aureus*; ST5, ST45, and ST239 have been detected in hospital-associated *S. aureus* infections; and ST1, ST8, ST30, and ST59 are often related to community-associated *S. aureus* infections (Bens et al., 2006; Cui and Li, 2009; Neela et al., 2009; Skov, 2009; Deleo et al., 2010). Generally, *spa* typing showed highly concordant with MLST. In the current study, MLST and *spa* typing enabled classification of pasteurized milk related *S. aureus* isolates into 11 types (ST188-t189, ST1-t177, ST9-899, ST12-t213, ST72-t3092, ST7-t091, ST5-t002, ST15-t085, ST25-t078, ST398-t034, and ST4069-t148). Most of these STs were detected in various of samples and relevant to a variety of clinical *S. aureus* infections (Krziwanek et al., 2009; Lee et al., 2011; Yu et al., 2012; Schaumburg et al., 2015). Therefore, the hazards of these strains for consumers cannot be ignored.

## Conclusion

To conclude, our results provide information regarding the genetic background of *S. aureus* strains obtained from pasteurized milk samples in China. To our knowledge, our study is the first systematic investigation of prevalence and contamination levels for *S. aureus* isolated from pasteurized milk samples in China. Different source strains showed different types in this study. These isolates had virulence potential, which half of them carried enterotoxin genes and infection-related molecular type. The resistance patterns can help determine more appropriate treatments for human infections in China. All the isolates had moderate or strong biofilm-production capability, and most of them harbored biofilm-related genes, indicating that these potentially virulent bacteria could persist in the food-manufacturing environment.

## Author Contributions

QW, JZ, JD, SW, and JH conceived and designed the experiments. JD, JH, and FZ performed the experiments. JD, SW, HW, and TL analyzed the data. XY, LX, YD, SZ, and JW contributed reagents, materials, and analysis tools. SW, JD, and QW contributed to the writing of the manuscript.

## Conflict of Interest Statement

The authors declare that the research was conducted in the absence of any commercial or financial relationships that could be construed as a potential conflict of interest.

## Footnotes

1 https://pubmlst.org/saureus/

2 https://spaserver2.ridom.de

3 https://pubmlst.org/bigsdb?db=pubmlst_saureus_isolates&page=profiles
