# Supplementary material for: Prevalence and Characterization of Staphylococcus aureus Isolated From Pasteurized Milk in China
Source: Front Microbiol. 2019 Apr 2;10:641. doi: 10.3389/fmicb.2019.00641 (PMC6454862; doi:10.3389/fmicb.2019.00641)
Supplement: Supplementary file 1 [file Data_Sheet_1.docx]

Supplementary Material

Prevalence and characterization of *Staphylococcus aureus* isolated from pasteurized milk in China

**Jingsha Dai#, Shi Wu#, Jiahui Huang#,Qingping Wu*, Feng Zhang, Jumei Zhang, Juan Wang, Yu Ding, Shuhong Zhang, Xiaojuan Yang, Tao Lei, Liang Xue, Haoming Wu**

*** Dr. Qingping Wu:** [**wuqp203@163.com**](mailto:wuqp203@163.com)

# Supplementary Figures and Tables

## Supplementary Figures

**
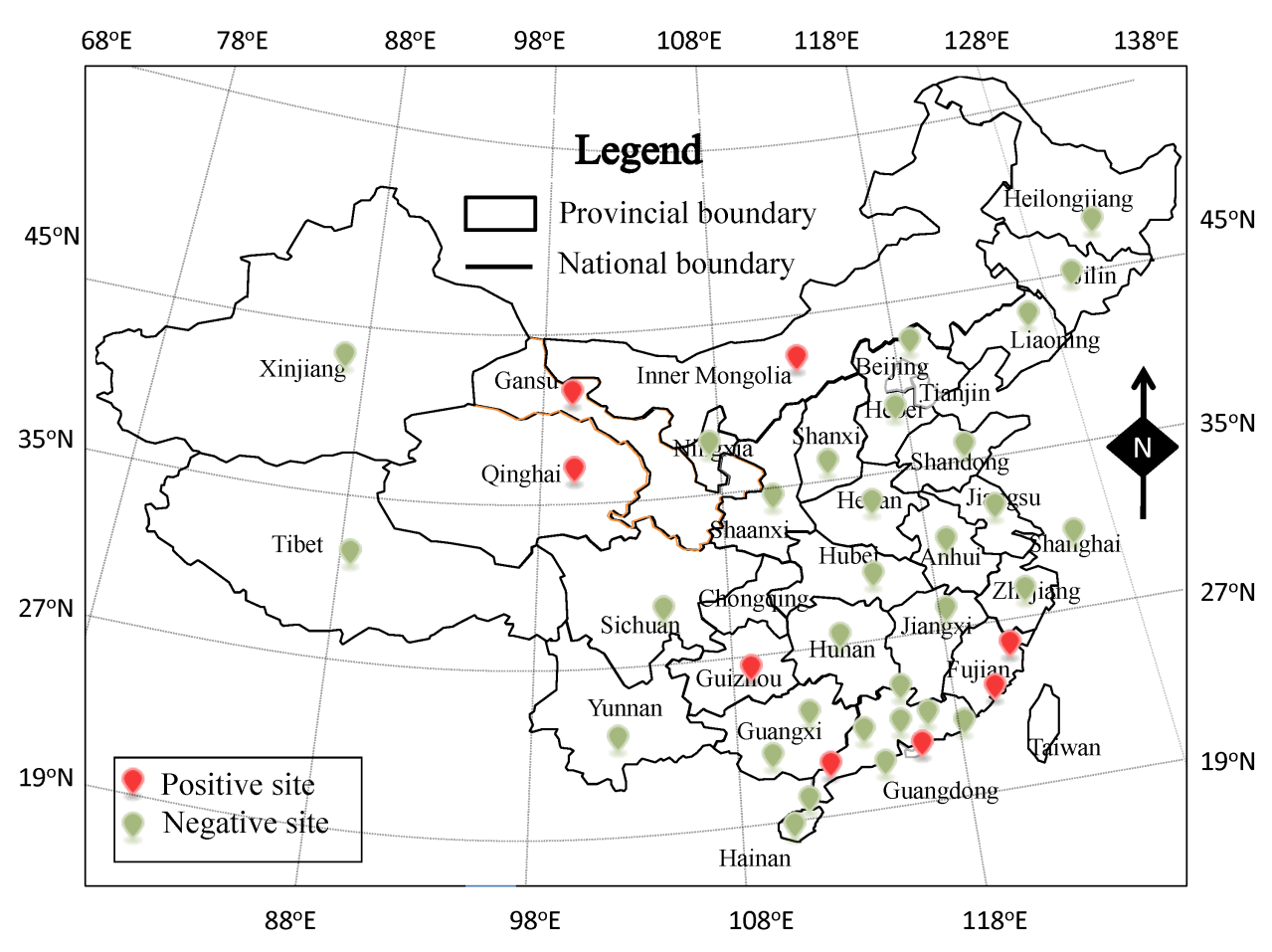
**

**Supplementary Figure 1.** The locations of the sampling sites for this study in China.

## Supplementary Tables

**Supplementary Table 1.** The information of samples in this study.

|  | NO. sample | Source | Location | Date in produced |
| --- | --- | --- | --- | --- |
| 1 | YXC8 | Supermarket | Guangzhou | N/A |
| 2 | YXJ32 | Fair | Guangzhou | N/A |
| 3 | YXJ33 | Fair | Guangzhou | 20130901 |
| 4 | LWC158 | Supermarket | Guangzhou | 20131007 |
| 5 | LWJ182 | Fair | Guangzhou | 20131008 |
| 6 | LWJ183 | Fair | Guangzhou | 20131009 |
| 7 | ZCC58 | Supermarket | Guangzhou | N/A |
| 8 | ZCC82 | Supermarket | Guangzhou | N/A |
| 9 | ZCC83 | Supermarket | Guangzhou | 2013727 |
| 10 | CHC108 | Supermarket | Guangzhou | 20130701 |
| 11 | CHJ132 | Fair | Guangzhou | 20130824 |
| 12 | CHJ133 | Fair | Guangzhou | 20130426 |
| 13 | PYC208 | Supermarket | Guangzhou | N/A |
| 14 | PYJ232 | Fair | Guangzhou | N/A |
| 15 | PYJ233 | Fair | Guangzhou | N/A |
| 16 | CHC1558 | Supermarket | Guangzhou | N/A |
| 17 | CHJ1582 | Fair | Guangzhou | N/A |
| 18 | CHJ1583 | Fair | Guangzhou | 20121229 |
| 19 | PYC1508 | Supermarket | Guangzhou | 20130319 |
| 20 | PYN1532 | Farmer`s market | Guangzhou | 20130319 |
| 21 | PYN1533 | Farmer`s market | Guangzhou | 20130319 |
| 22 | LWC1458 | Supermarket | Guangzhou | N/A |
| 23 | LWN1482 | Farmer`s market | Guangzhou | N/A |
| 24 | LWN1483 | Farmer`s market | Guangzhou | 20130312 |
| 25 | YXC1408 | Supermarket | Guangzhou | 20130301 |
| 26 | YXJ1432 | Fair | Guangzhou | N/A |
| 27 | YXJ1433 | Fair | Guangzhou | 20130301 |
| 28 | ZCC1608 | Supermarket | Guangzhou | 20130408 |
| 29 | ZCJ1632 | Fair | Guangzhou | 20130321 |
| 30 | ZCJ1633 | Fair | Guangzhou | 20130212 |
| 31 | HYC458 | Supermarket | Guangzhou | 20120302 |
| 32 | HYJ481 | Fair | Heyuan | 20110609 |
| 33 | HYN496 | Farmer`s market | Heyuan | 20120320 |
| 34 | STC408 | Supermarket | Shantao | 20120215 |
| 35 | STJ426 | Fair | Shantao | 20120212 |
| 36 | STN441 | Farmer`s market | Shantao | 20111215 |
| 37 | SGC308 | Supermarket | Shaoguan | 20111219 |
| 38 | SGJ331 | Fair | Shaoguan | 20110920 |
| 39 | SGN346 | Farmer`s market | Shaoguan | 20120.22 |
| 40 | SZC258 | Supermarket | Shenzhen | 20111209 |
| 41 | SZJ276 | Fair | Shenzhen | 20111207 |
| 42 | SZN291 | Farmer`s market | Shenzhen | 20111207 |
| 43 | ZJC358 | Supermarket | Zhanjiang | 20120105 |
| 44 | ZJJ376 | Fair | Zhanjiang | 20120103 |
| 45 | ZJN391 | Farmer`s market | Zhanjiang | 20120104 |
| 46 | HYC1858 | Supermarket | Heyuan | N/A |
| 47 | HYJ1882 | Fair | Heyuan | N/A |
| 48 | HYJ1883 | Fair | Heyuan | N/A |
| 49 | STC1708 | Supermarket | Shantou | N/A |
| 50 | STJ1732 | Fair | Shantou | N/A |
| 51 | STJ1733 | Fair | Shantou | N/A |
| 52 | SGC1808 | Supermarket | Shaoguan | N/A |
| 53 | SGJ1832 | Fair | Shaoguan | N/A |
| 54 | SGJ1833 | Fair | Shaoguan | N/A |
| 55 | SZC1658 | Supermarket | Shenzhen | N/A |
| 56 | SZJ1682 | Fair | Shenzhen | N/A |
| 57 | SZJ1683 | Fair | Shenzhen | N/A |
| 58 | ZJC1758 | Supermarket | Zhanjiang | 20130413 |
| 59 | ZJJ1782 | Fair | Zhanjiang | 20130526 |
| 60 | ZJJ1783 | Fair | Zhanjiang | N/A |
| 61 | BHC608 | Supermarket | Beihai | 20120506 |
| 62 | BHJ626 | Fair | Beihai | 20120426 |
| 63 | BHN641 | Farmer`s market | Beihai | 20120520 |
| 64 | FZC708 | Supermarket | Fuzhou | 20120624 |
| 65 | FZJ726 | Fair | Fuzhou | 20120702 |
| 66 | FZN741 | Farmer`s market | Fuzhou | 20120612 |
| 67 | HKC508 | Supermarket | Haikou | 20120503 |
| 68 | HKJ526 | Fair | Haikou | 20120501 |
| 69 | HKN541 | Farmer`s market | Haikou | 20120501 |
| 70 | NNC658 | Supermarket | Nanning | 20120702 |
| 71 | NNJ676 | Fair | Nanning | 20120702 |
| 72 | NNN691 | Farmer`s market | Nanning | 20120702 |
| 73 | SYC558 | Supermarket | Sanya | 20120517 |
| 74 | SYJ576 | Fair | Sanya | 20120510 |
| 75 | SYN591 | Farmer`s market | Sanya | 20120422 |
| 76 | XMC758 | Supermarket | Xiamen | 20120801 |
| 77 | XMJ776 | Fair | Xiamen | 20120624 |
| 78 | XMN791 | Farmer`s market | Xiamen | 20120802 |
| 79 | BJC1308 | Supermarket | Beijing | 20121215 |
| 80 | BJJ1327 | Fair | Beijing | 20121217 |
| 81 | BJN1342 | Farmer`s market | Beijing | 20121217 |
| 82 | CDC1008 | Supermarket | Chengdu | 20120925 |
| 83 | CDJ1027 | Fair | Chengdu | 20121001 |
| 84 | CDN1042 | Farmer`s market | Chengdu | 20121004 |
| 85 | HEBC1158 | Supermarket | Haerbin | 20121120 |
| 86 | HEBJ1177 | Fair | Haerbin | 20121120 |
| 87 | HEBN1192 | Farmer`s market | Haerbin | 20121120 |
| 88 | HFC858 | Supermarket | Hefei | N/A |
| 89 | HFJ877 | Fair | Hefei | N/A |
| 90 | HFJ892 | Fair | Hefei | N/A |
| 91 | JNC1358 | Supermarket | Jinan | 20121220 |
| 92 | JNJ1377 | Fair | Jinan | 20121224 |
| 93 | JNN1392 | Farmer`s market | Jinan | 20121224 |
| 94 | KMC1058 | Supermarket | Kunming | 20121104 |
| 95 | KMJ1077 | Fair | Kunming | N/A |
| 96 | KMN1092 | Farmer`s market | Kunming | N/A |
| 97 | LZC1108 | Supermarket | Lanzhou | 20121109 |
| 98 | LZJ1127 | Fair | Lanzhou | 20121111 |
| 99 | LZN1142 | Farmer`s market | Lanzhou | 20121111 |
| 100 | NCC908 | Supermarket | Nanchang | 20120922 |
| 101 | NCJ927 | Fair | Nanchang | 20120921 |
| 102 | NCN942 | Farmer`s market | Nanchang | 20120920 |
| 103 | SHC808 | Supermarket | Shanghai | 20120904 |
| 104 | SHJ827 | Fair | Shanghai | 20120904 |
| 105 | SHN842 | Farmer`s market | Shanghai | 20120904 |
| 106 | TYC1258 | Supermarket | Taiyuan | 20121211 |
| 107 | TYJ1277 | Fair | Taiyuan | 20121124 |
| 108 | TYN1292 | Farmer`s market | Taiyuan | 20121206 |
| 109 | WHC958 | Supermarket | Wuhan | 20121007 |
| 110 | WHJ977 | Fair | Wuhan | N/A |
| 111 | WHN992 | Farmer`s market | Wuhan | 20121007 |
| 112 | XAC1208 | Supermarket | Xi'an | 20121129 |
| 113 | XAJ1227 | Fair | Xi'an | 20121129 |
| 114 | XAN1242 | Farmer`s market | Xi'an | 20121129 |
| 115 | BHC2358 | Supermarket | Beihai | 20131009 |
| 116 | BHJ2382 | Fair | Beihai | 20131023 |
| 117 | BHJ2383 | Fair | Beihai | 20131023 |
| 118 | FZC2208 | Supermarket | Fuzhou | N/A |
| 119 | FZJ2232 | Fair | Fuzhou | N/A |
| 120 | FZJ2233 | Fair | Fuzhou | N/A |
| 121 | HKC2408 | Supermarket | Haikou | 20131219 |
| 122 | HKJ2432 | Fair | Haikou | 20131221 |
| 123 | HKJ2433 | Fair | Haikou | 20130926 |
| 124 | NNC2258 | Supermarket | Nanning | N/A |
| 125 | NNJ2282 | Fair | Nanning | N/A |
| 126 | NNJ2283 | Fair | Nanning | N/A |
| 127 | SYC2458 | Supermarket | Sanya | 20140102 |
| 128 | SYJ2482 | Fair | Sanya | N/A |
| 129 | SYJ2483 | Fair | Sanya | N/A |
| 130 | XMC2308 | Supermarket | Xiamen | 20131126 |
| 131 | XMJ2332 | Fair | Xiamen | 20131126 |
| 132 | XMJ2333 | Fair | Xiamen | 20131126 |
| 133 | BJC2058 | Supermarket | Beijing | N/A |
| 134 | BJJ2082 | Fair | Beijing | N/A |
| 135 | BJJ2083 | Fair | Beijing | N/A |
| 136 | HEBC1958 | Supermarket | Haerbin | N/A |
| 137 | HEBJ1982 | Fair | Haerbin | N/A |
| 138 | HEBJ1983 | Fair | Haerbin | N/A |
| 139 | JNC2008 | Supermarket | Jinan | 20130728 |
| 140 | JNJ2027 | Fair | Jinan | 20130723 |
| 141 | JNJ2042 | Fair | Jinan | 20130721 |
| 142 | LZC2158 | Supermarket | Lanzhou | 20130625 |
| 143 | LZJ2177 | Fair | Lanzhou | 20130801 |
| 144 | LZJ2192 | Fair | Lanzhou | 20130816 |
| 145 | TYC2108 | Supermarket | Taiyuan | 20130815 |
| 146 | TYJ2132 | Fair | Taiyuan | 20130818 |
| 147 | TYJ2133 | Fair | Taiyuan | 20130817 |
| 148 | XAC1908 | Supermarket | Xi'an | N/A |
| 149 | XAJ1932 | Fair | Xi'an | N/A |
| 150 | XAJ1933 | Fair | Xi'an | N/A |
| 151 | NCC2508 | Supermarket | Nnachang | 20140301 |
| 152 | NCJ2532 | Fair | Nnachang | 20140301 |
| 153 | NCJ2533 | Fair | Nnachang | 20140301 |
| 154 | CDC2558 | Supermarket | Chengdu | 140312 |
| 155 | CDJ2582 | Fair | Chengdu | 140309 |
| 156 | CDJ2583 | Fair | Chengdu | 140311 |
| 157 | HFC2608 | Supermarket | Hefei | 20140211 |
| 158 | HFJ2632 | Fair | Hefei | 20141119 |
| 159 | HFJ2633 | Fair | Hefei | 20131216 |
| 160 | WHC2658 | Supermarket | Wuhan | 20140408 |
| 161 | WHJ2682 | Fair | Wuhan | 20140408 |
| 162 | WHJ2683 | Fair | Wuhan | 20140409 |
| 163 | SHC2708 | Supermarket | Shanghai | 20140423 |
| 164 | SHJ2732 | Fair | Shanghai | 20140423 |
| 165 | SHJ2733 | Fair | Shanghai | 20140422 |
| 166 | KMC2758 | Supermarket | Kunming | 20140506 |
| 167 | KMJ2782 | Fair | Kunming | 20140505 |
| 168 | KMJ2783 | Fair | Kunming | 20140505 |
| 169 | CSC2808 | Supermarket | Changsha | N/A |
| 170 | CSJ2832 | Fair | Changsha | N/A |
| 171 | CSJ2833 | Fair | Changsha | N/A |
| 172 | HZC2858 | Supermarket | Hangzhou | 20150716 |
| 173 | HZJ2882 | Fair | Hangzhou | 20150715 |
| 174 | HZJ2883 | Fair | Hangzhou | 20150716 |
| 175 | GYC2908 | Supermarket | Guiyang | N/A |
| 176 | GYJ2932 | Fair | Guiyang | 20150723 |
| 177 | GYJ2933 | Fair | Guiyang | 20150714 |
| 178 | CCC2958 | Supermarket | Changchun | 20150804 |
| 179 | CCJ2982 | Fair | Changchun | 20150804 |
| 180 | CCJ2983 | Fair | Changchun | 20150802 |
| 181 | XNC3008 | Supermarket | Xining | 20150819 |
| 182 | XNJ3032 | Fair | Xining | 20150810 |
| 183 | XNJ3033 | Fair | Xining | 20150819 |
| 184 | YCC3058 | Supermarket | Yinchuan | 20150824 |
| 185 | YCJ3082 | Fair | Yinchuan | 20150822 |
| 186 | YCJ3083 | Fair | Yinchuan | 20150823 |
| 187 | HHHTC3108 | Supermarket | Huhehaote | 20150904 |
| 188 | HHHTJ3132 | Fair | Huhehaote | 20150828 |
| 189 | HHHTJ3133 | Fair | Huhehaote | 20150905 |
| 190 | SYC3158 | Supermarket | Shenyang | 20150910 |
| 191 | SYJ3182 | Fair | Shenyang | 20150912 |
| 192 | SYJ3183 | Fair | Shenyang | 20150913 |
| 193 | NJC3208 | Supermarket | Nanjing | 20150919 |
| 194 | NJJ3232 | Fair | Nanjing | 20150920 |
| 195 | NJJ3233 | Fair | Nanjing | 20150918 |
| 196 | SJZC3258 | Supermarket | Shijiazhuang | 20151013 |
| 197 | SJZJ3282 | Fair | Shijiazhuang | 20151011 |
| 198 | SJZJ3283 | Fair | Shijiazhuang | 20151013 |
| 199 | ZZC3308 | Supermarket | Zhengzhou | 20150818 |
| 200 | ZZJ3332 | Fair | Zhengzhou | 20151018 |
| 201 | ZZJ3333 | Fair | Zhengzhou | 20151007 |
| 202 | LSC3358 | Supermarket | Lasa | 20150721 |
| 203 | LSJ3382 | Fair | Lasa | 20151024 |
| 204 | LSJ3383 | Fair | Lasa | 20150910 |
| 205 | WLMQC3408 | Supermarket | Wulumuqi | 20151123 |
| 206 | WLMQJ3432 | Fair | Wulumuqi | 20151110 |
| 207 | WLMQJ3433 | Fair | Wulumuqi | 20151123 |
| 208 | AMC3458 | Supermarket | Macao | 20151211 |
| 209 | AMJ3482 | Fair | Macao | 20151213 |
| 210 | AMJ3483 | Fair | Macao | 20151211 |
| 211 | HKC3508 | Supermarket | Hongkong | N/A |
| 212 | HKJ3532 | Fair | Hongkong | N/A |
| 213 | HKJ3533 | Fair | Hongkong | N/A |
| 214 | CSC3558 | Supermarket | Changsha | 20160109 |
| 215 | CSJ3582 | Fair | Changsha | 20160107 |
| 216 | CSJ3583 | Fair | Changsha | 20160110 |
| 217 | GYC3608 | Supermarket | Guiyang | 20160117 |
| 218 | GYJ3632 | Fair | Guiyang | 20160115 |
| 219 | GYJ3633 | Fair | Guiyang | 20160119 |
| 220 | HZC3658 | Supermarket | Hangzhou | 20160222 |
| 221 | HZJ3682 | Fair | Hangzhou | 20160222 |
| 222 | HZJ3683 | Fair | Hangzhou | 20160223 |
| 223 | NJC3708 | Supermarket | Nanjing | N/A |
| 224 | NJJ3732 | Fair | Nanjing | N/A |
| 225 | NJJ3733 | Fair | Nanjing | N/A |
| 226 | SJZC3758 | Supermarket | Shijiazhuang | 20160306 |
| 227 | SJZJ3782 | Fair | Shijiazhuang | 20160226 |
| 228 | SJZJ3783 | Fair | Shijiazhuang | 20160304 |
| 229 | SYC3808 | Supermarket | Shenyang | 20160316 |
| 230 | SYJ3832 | Fair | Shenyang | 20160316 |
| 231 | SYJ3833 | Fair | Shenyang | 20160306 |
| 232 | YCC3858 | Supermarket | Yinchuan | 20160221 |
| 233 | YCJ3882 | Fair | Yinchuan | 20160307 |
| 234 | YCJ3883 | Fair | Yinchuan | 20160325 |
| 235 | ZZC3908 | Supermarket | Zhengzhou | 20160210 |
| 236 | ZZJ3932 | Fair | Zhengzhou | 20151018 |
| 237 | ZZJ3933 | Fair | Zhengzhou | 20151007 |
| 238 | XNC3958 | Supermarket | Xining | 20160325 |
| 239 | XNJ3982 | Fair | Xining | 20160406 |
| 240 | XNJ3983 | Fair | Xining | 20160413 |
| 241 | HHHTC4008 | Supermarket | Huhehaote | 20160421 |
| 242 | HHHTJ4032 | Fair | Huhehaote | 20160416 |
| 243 | HHHTJ4033 | Fair | Huhehaote | 20160413 |
| 244 | CCC4058 | Supermarket | Changchun | N/A |
| 245 | CCJ4082 | Fair | Changchun | N/A |
| 246 | CCJ4083 | Fair | Changchun | N/A |
| 247 | AMC4108 | Supermarket | Macao | N/A |
| 248 | AMJ4132 | Fair | Macao | 20151213 |
| 249 | AMJ4133 | Fair | Macao | 20151211 |
| 250 | XGC4158 | Supermarket | Hongkong | 2016.5.26 |
| 251 | XGJ4182 | Fair | Hongkong | N/A |
| 252 | XGJ4183 | Fair | Hongkong | N/A |
| 253 | WLMQC4208 | Supermarket | Wulumuqi | 20160615 |
| 254 | WLMQJ4232 | Fair | Wulumuqi | 20160513 |
| 255 | WLMQJ4233 | Fair | Wulumuqi | 20160513 |
| 256 | LSC4258 | Supermarket | Lasa | 20160328 |
| 257 | LSJ4282 | Fair | Lasa | 20160503 |
| 258 | LSJ4283 | Fair | Lasa | 20160430 |

**Supplementary Table 2.** Sequences of primers used for detection of virulence genes and biofilm-related genes in this study

| Gene target | Primer sequences (5’-3’) | Product sizes(bp) | References |
| --- | --- | --- | --- |
| *pvl* | F: ATCATTAGGTAAAATGTCTGGACATGATCCA | 433bp | X. Wang et al. (2012) |
|  | R: GCATCAASTGTATTGGATAGCAAAAGC |  |  |
| *tsst* | F: AAG CCC TTT GTT GCT TGC G | 447bp | Avanish K. Varshney et al. (2009) |
|  | R:ATC GAA CTT TGG CCC ATA CTT T |  |  |
| *sea* | F: GAAAAAAGTCTGAATTGCAGGGAACA | 561bp | I. Ote et al. (2011) |
|  | R: CAAATAAATCGTAATTAACCGAAGGTTC |  |  |
| *seb* | TCGCATCAAACTGACAAACG | 477bp | Avanish K. Varshney et al. (2009) |
|  | GCAGGTACTCTATAAGTGCCTGC |  |  |
| *sec* | F: AGATTTAGCAAAGAAGTACAAAGATG | 490bp | Avanish K. Varshney et al. (2009) |
|  | R: AAGGTGGACTTCTATCTTCACACTT |  |  |
| *sed* | F: CTA GTT TGG TAA TAT CTC CT | 319bp | I. Ote et al. (2011) |
|  | R: TA ATG CTA TAT CTT ATA GGG |  |  |
| *see* | F: ACCGATTGACCGAAGAAAAA | 264bp | Avanish K. Varshney et al. (2009) |
|  | R: ATTGCCCTTGAGCATCAAAC |  |  |
| *seg* | F: AGAATTAGCTAACAATTATAAAGATAAAAAAG | 496bp | Avanish K. Varshney et al. (2009) |
|  | R: TCAGTGAGTATTAAGAAATACTTCCAT |  |  |
| *she* | F: TGATTTAGCTCAGAAGTTTAAAAATAAAAATG | 466bp | Avanish K. Varshney et al. (2009) |
|  | R: TTTCTTAGTATATAGATTTACATCAATATG |  |  |
| *sei* | F: TGGAACAGGACAAGCTGAAA | 529bp | Avanish K. Varshney et al. (2009) |
|  | R: TGTTTGCCATTAACCCAAAG |  |  |
| *sej* | F: ATGAAAAAAACAATATTTATACTGATTTTCTCCC | 807bp | Avanish K. Varshney et al. (2009) |
|  | R: TCTACAGAACCAAAGGTAGACTTATTAATAC |  |  |
| *sek* | F: ATGAATCTTATGATTTAATTTCAGAATCAA | 545bp | Avanish K. Varshney et al. (2009) |
|  | R: ATTTATATCGTTTCTTTATAAGAAATATCG |  |  |
| *sel* | F: ATGAAAAAAAGATTATTATTTGTAATTGTTATTAC | 723bp | Avanish K. Varshney et al. (2009) |
|  | R: ATCATCTTTTTGAAATTTCGACATCTAG |  |  |
| *sem* | F: ATGAAAAGAATACTTATCATTGTTGTTTTATTG | 720bp | Avanish K. Varshney et al. (2009) |
|  | R: CTTCAACTTTCGTCCTTATAAGATATTTC |  |  |
| *sen* | F: ATAAAAAATATTAAAAAGCTTATGAGATTGTTC | 777bp | Avanish K. Varshney et al. (2009) |
|  | R: ACTTAATCTTTATATAAAAATACATCAATATG |  |  |
| *seo* | F: TATGTAGTGTAAACAATGCATATGCA | 685bp | Avanish K. Varshney et al. (2009) |
|  | R: TCTATTGTTTTATTATCATTATAAATTTGCAAAT |  |  |
| *sep* | F: TTAGACAAACCTATTATCATAATGGAAGT | 618bp | Avanish K. Varshney et al. (2009) |
|  | R: TATATAAATATATATCAATATGCATATTTTTAGACT |  |  |
| *seq* | F: GGAAAATACACTTTATATTCACAGTTTCA | 539bp | Avanish K. Varshney et al. (2009) |
|  | R: ATTTATTCAGTTTTCTCATATGAAATCTC |  |  |
| *ser* | F: AGCGGTAATAGCAGAAAATG | 363bp | Avanish K. Varshney et al. (2009) |
|  | R: TCTTGTACCGTAACCGTTTT |  |  |
| *seu* | F: AATGGCTCTAAAATTGATGG | 215bp | Avanish K. Varshney et al. (2009) |
|  | R: ATTTGATTTCCATCATGCTC |  |  |
| *clfA* | F: ATTGGCGTGGCTTCAGTGCT | 292bp | L. Li et al. (2012) |
|  | R: CGTTTCTTCCGTAGTTGCATTTG |  |  |
| *clfB* | F: ACATCAGTAATAGTAGGGGGCAAC | 205bp | L. Li et al. (2012) |
|  | R: TTCGCACTGTTTGTGTTTGCAC |  |  |
| *fnbA* | F: GATACAAACCCAGGTGGTGG | 191bp | T. Zmantar et al. (2008) |
|  | R: TGTGCTTGACCATGCTCTTC |  |  |
| *fnbB* | F: ACGCTCAAGGCGACGGCAAAG | 197bp | Elizabet A.L. et al. (2016) |
|  | R: ACCTTCTGCATGACCTTCTGCACCT |  |  |
| *fib* | F: CGTCAACAGCAGATGCGAGCG | 239bp | Atshan, S.S. et al. (2013) |
|  | R: TGCATCAGTTTTCGCTGCTGGTTT |  |  |
| *cna* | F: AAAGCGTTGCCTAGTGGAGAC | 192bp | T. Zmantar et al. (2008) |
|  | R: AGTGCCTTCCCAAACCTTTT |  |  |
| *icaA* | F: TATACCTTTCTTCGATGTCG | 561bp | P. Vasudevan, et al.(2003) |
|  | R: CTTTCGTTATAACAGGCAAG |  |  |
| *icaD* | F: ATGGTCAAGCCCAGACAGAG | 198bp | Arciola C R, et al.(2001) |
|  | R: CGTGTTTTCAACATTTAATGCAA |  |  |
| *icaC* | F: CTTGGGTATTTGCACGCATT | 209bp | Elizabet A.L. et al. (2016) |
|  | R: GCAATATCATGCCGACACCT |  |  |
| *bap* | F: CCCTATATCGAAGGTGTAGAATTGCAC | 971bp | C. Cucarella et al. (2001) |
|  | R: GCTGTTGAAGTTAATACTGTACCTGC |  |  |
| *eno* | F: ACGTGCAGCAGCTGACT | 302bp | Sandra Renata et al. (2015) |
|  | R: CAACAGCATCTTCAGTACCTTC |  |  |
